# When Do We Recover from Loneliness: The Role of Green Consumption and Social Value

**DOI:** 10.3390/bs16030427

**Published:** 2026-03-16

**Authors:** Meiling Yin, Mingyeong Jeon

**Affiliations:** School of Business, Sejong University, Seoul 05006, Republic of Korea; mlyin88@sejong.ac.kr

**Keywords:** loneliness, prosocial behavior, green consumption, emotional recovery

## Abstract

While prior research has shown that loneliness has negative consequences on individuals’ mental and physical health, little is known about when and how individuals emotionally recover from it. This study investigates whether individuals’ engagement in green consumption alters the extent to which they emotionally recover from loneliness. Across behavioral experiments, we show that green consumption offers emotional benefit for those experiencing loneliness through their perceived social value. This research advances the theoretical understanding of the effectiveness of green consumption in emotional recovery from loneliness. Moreover, we provide practical guidance for marketers to design advertisements and campaigns that emphasize the social and environmental benefits of green consumption.

## 1. Introduction

In contemporary society, loneliness is increasingly recognized not merely as a transient emotional state but as a pervasive psychological condition, often described as the “disease of modern society.” Accordingly, approximately one-third of adults worldwide reported experiencing feelings of loneliness (Statista Research Department, 2025). Loneliness is not only emotionally distressing but also associated with serious consequences for mental and psychological health. For instance, it is closely related to heightened levels of depression, anxiety, and perceived social isolation (Ni et al., 2020; L. Z. Li & Wang, 2020; Yang et al., 2023). Therefore, exploring methods to cope with and overcome loneliness has become a critical concern in modern society.

Although increased social interaction, such as going out and engaging with others, has been suggested as an effective means of reducing loneliness (Kellezi et al., 2019; Snyder & Newman, 2019; Lapena et al., 2020), such activities do not always succeed in alleviating lonely individuals’ emotional distress. In fact, loneliness is frequently accompanied by self-preservation motives, which may prompt individuals to withdraw from direct social engagement to avoid potential rejection or discomfort (Masi et al., 2011; Malon et al., 2024). This paradox suggests that lonely individuals may seek alternative coping strategies that do not require direct interpersonal interaction.

Consumption behaviors have been identified as one such compensatory resource, providing emotional comfort and symbolic meaning in the absence of social support (Mittal & Silvera, 2018). Prior research suggests that individuals experiencing psychological deficits, such as loneliness, may engage in purchasing behaviors as a form of symbolic self-repair, yet reliance on consumption for mood regulation can also lead to maladaptive patterns such as excessive or compulsive buying (Ridgway et al., 2008; Mandel et al., 2017). Accordingly, the psychological consequences of consumption may depend on the motivations underlying the behavior. When consumption is driven primarily by extrinsic motives, such as materialistic values (Dittmar, 2005), reliance on purchasing as a coping strategy may become maladaptive. In contrast, behaviors motivated by intrinsic values are more likely to foster meaningful psychological benefits (Kasser & Ryan, 1996; Ryan & Deci, 2000).

Building on this perspective, recent research has begun to consider whether green consumption may serve as a potential emotional coping strategy (Gao & Mattila, 2016; Guo et al., 2020). Importantly, green consumption is not merely an everyday independent activity; rather, eco-friendly purchasing carries socially valued meanings, signaling responsibility and prosocial identity (Griskevicius et al., 2010; Sweeney & Soutar, 2001). As such, engaging in green consumption may allow lonely individuals to experience symbolic reassurance and a sense of connection without the interpersonal risks associated with direct social engagement. Despite its potential importance, the role of green consumption in facilitating emotional recovery from loneliness remains largely overlooked. To address this gap, the present study investigates green consumption as a moderating factor in the loneliness-recovery process. Across a series of behavioral experiments, we examine whether engaging in green consumption helps lonely individuals alleviate feelings of loneliness and assess social value as a key psychological mechanism underlying this effect.

In so doing, our study makes several theoretical contributions to the literature on consumer behavior and psychology. First, we extend loneliness and belongingness theory by demonstrating that recovery from loneliness may occur not only through direct interpersonal interaction but also through symbolic pathways that restore perceived social connectedness (Baumeister & Leary, 1995; Hawkley & Cacioppo, 2010). Specifically, feelings of loneliness are not alleviated solely through direct interaction; rather, they may also be mitigated through the symbolic forms of prosocial engagement that foster a sense of belonging. In this vein, we position green consumption as a low-risk form of prosocial behavior that allows lonely individuals to psychologically reconnect with others and regain a sense of social belonging, even when direct interaction is less accessible. Thus, this research advances understanding of how lonely individuals cope with social disconnection when socially demanding behaviors are difficult to pursue.

Second, we contribute to the sustainable consumption literature by identifying green consumption as a distinct form of socially valued behavior that yields psychological benefits beyond environmental outcomes. Rather than treating eco-friendly purchasing as merely an ethical marketplace choice, our findings suggest that it can function as an emotional coping strategy through which individuals reaffirm social worth. Third, by introducing perceived social value as a mediating psychological mechanism, this study clarifies why and when green consumption facilitates emotional recovery. This process-oriented perspective deepens theoretical insights into the affective consequences of sustainable consumption and highlights social value restoration as a key psychological mechanism linking marketplace behavior to emotional well-being. Finally, our work offers practical implications by suggesting that firms may incorporate social value cues into green marketing strategies, not only to promote sustainable product adoption but also to support consumers’ psychological need for social recognition and connection.

## 2. Literature Review

### 2.1. Loneliness

Loneliness is an inherently subjective and intangible emotion; it is generally recognized as a negative state that individuals seek to avoid (Stein & Tuval-Mashiach, 2015). In this study, loneliness is defined as perceived social isolation, disconnection, and perceived deficits in social relationships (Cacioppo et al., 2006). This definition aligns with Peplau and Perlman’s (1982) framework, which describes loneliness as a negative feeling triggered when one’s social relationships are perceived as insufficient in quantity or quality.

Beyond its prevalence, a particularly important question is whether individuals are able to recover emotionally from loneliness once it is experienced. Loneliness often disrupts the emotional regulation process and fosters persistent negative thinking, which can undermine individuals’ capacity to restore emotional balance (Okan et al., 2023). As a result, lonely individuals may struggle to regain positive affect and are more likely to engage in maladaptive behaviors that further impede emotional restoration. Empirical evidence links loneliness to smoking, physical inactivity, and problematic internet use (Shankar et al., 2011; Savolainen et al., 2020); heightened impulsivity reflected in excessive purchasing and materialism (Shrum et al., 2023); and poorer functioning in work contexts, including reduced creativity and productivity (Çinar & Toker, 2019).

Moreover, loneliness is consistently associated with broader psychological distress, including increased risks of anxiety (Moore & Schultz, 1983), depression (Lau et al., 1999), and even suicidal ideation and behavior (Schinka et al., 2013). These findings underscore that loneliness is not merely a transient negative emotional state but a profound psychological condition that undermines individuals’ emotional resilience. Supporting this view, Caple et al. (2023) highlight that loneliness is closely linked to poorer mental health recovery outcomes, suggesting that loneliness can hinder individuals’ ability to regain emotional well-being. In particular, loneliness may reduce coping resources and weaken one’s ability to regulate and recover from adverse emotional experiences. Given this established vulnerability, we expect that loneliness will reduce emotional recovery relative to non-lonely states.

**H1.**  *Individuals exposed to the loneliness priming condition will exhibit lower emotional recovery compared to those in the control condition.*

### 2.2. Green Consumption Behavior

Green products are items that contribute to environmental conservation, such as organic and energy-saving products. Green consumption behavior (GCB) refers to environmentally friendly actions, including the intentional purchase or use of eco-friendly products, which can signal one’s prosocial nature (Johnstone & Hooper, 2016; Guo et al., 2020). Prior research has shown that prosocial behavior may reduce loneliness by fostering social bonds (Dunn et al., 2008; Martela & Ryan, 2016; Y. Lee et al., 2024). However, such evidence largely assumes that prosocial acts operate through direct social interaction, which may not always be accessible to lonely individuals (Hawkley & Cacioppo, 2010).

Importantly, lonely individuals often have heightened sensitivity and self-protective motives, which reduce their willingness to engage in socially demanding forms of prosocial behavior (Masi et al., 2011; Malon et al., 2024). Supporting this view, Yin and Lee (2023) demonstrate that loneliness cues can decrease engagement in direct prosocial helping because lonely individuals perceive social situations as more threatening and potentially rejecting. As a result, rather than seeking connection through interpersonal helping, lonely individuals may turn to alternative ways of finding meaningful connection with the outside world, such as through nature or pro-environmental actions (Qu et al., 2025). In this regard, green consumption represents a theoretically distinct form of prosocial engagement.

Green consumption may help lonely individuals recover more effectively from loneliness-related negative emotions. Loneliness is closely tied to deficits in perceived meaning, both in their experiences and daily life (Tam & Chan, 2019). Importantly, consumption experiences that enhance meaning have been shown to alleviate loneliness by restoring a sense of purpose in life (Wang et al., 2021). Extending this perspective, eco-friendly purchasing can also function as a coping-oriented response to negative emotional states (Barbarossa & De Pelsmacker, 2016), suggesting that green consumption may provide emotional benefits beyond its environmental impact. Recent evidence further indicates that green choices can facilitate well-being and emotional recovery among socially excluded individuals (Khan, 2024).

These findings imply that green consumption represents a meaningful behavioral resource that may help individuals manage loneliness-related distress. Since green consumption is often perceived as a socially positive contribution (Griskevicius et al., 2010), engaging in such behavior may enable lonely individuals to experience psychological reassurance and emotional comfort even in the absence of direct interpersonal contact. Accordingly, green consumption may serve as a low-risk strategy through which lonely individuals can regain emotional well-being without requiring immediate social exposure. Therefore, we expect that green consumption will buffer the detrimental effect of loneliness on emotional recovery by offering a meaningful form of engagement.

**H2.**  *Individuals exposed to the loneliness priming condition (vs. the control condition) exhibit higher emotional recovery when engaging in green consumption than when they do not.*

### 2.3. Social Value as a Psychological Mechanism for Emotional Recovery

Social value refers to the perceived extent to which certain actions allow individuals to gain acceptance and approval from society (Sweeney & Soutar, 2001; Griskevicius et al., 2010). It reflects individuals’ desire to maintain a positive social image and to feel recognized as a valued member of the social world. Importantly, socially valued experiences are closely tied to emotional well-being because they help fulfill belongingness needs and reinforce positive self-regard (Baumeister & Leary, 1995). Prior studies further suggest that green consumption can enhance perceptions of being socially valued, indicating that eco-friendly behavior carries symbolic social significance beyond its environmental impact (Tezer & Bodur, 2020; Nguyen & Johnson, 2020).

The perceived social value is particularly relevant in the context of loneliness. Loneliness reflects not only a lack of social connection but also a diminished social worth (Peplau & Perlman, 1982; Cacioppo et al., 2006). When individuals feel lonely, they may interpret their social environment as less supportive and perceive themselves as less valued by others. As a result, lonely individuals often become highly sensitive to cues of rejection and exclusion, which further reinforces their sense of being socially undervalued (Hawkley & Cacioppo, 2010). This diminished social value can make it more difficult for them to regulate negative emotions and achieve emotional recovery.

Green consumption may therefore play an important role in restoring this threatened sense of social value. For instance, Gonçalves et al. (2016), drawing on the consumption values perspective, show that green purchasing emerges from different configurations of functional, emotional, and social values, suggesting that individuals often engage in eco-friendly consumption for reasons that extend beyond ecological outcomes. This perspective indicates that green consumption can serve an important socio-emotional function. By engaging in environmentally responsible consumption, individuals may perceive themselves as prosocial and socially responsible members of the broader community, thereby gaining a sense of social approval and worth (Mazar & Zhong, 2010; White et al., 2019). In this way, green consumption may offer lonely individuals psychological reassurance and help restore their diminished sense of social value, even in the absence of direct interpersonal interaction.

Critically, this restoration of perceived social value may explain why green consumption facilitates emotional recovery among lonely individuals. When individuals are able to reaffirm their social worth through eco-friendly choices, they may experience greater psychological reassurance that helps them rebound from loneliness-related distress (Gao & Mattila, 2016; Shrum et al., 2023). Emerging evidence further suggests that loneliness can motivate pro-environmental behavior as a compensatory strategy, through which individuals seek substitute sources of satisfaction (Qu et al., 2025). In line with this view, purchasing green products has been shown to facilitate emotional well-being among socially excluded individuals by restoring meaning and self-worth (Khan, 2024). Supporting this view, Sun et al. (2022) found that consumers engage in green products in order to gain emotional benefits such as psychological well-being (Sun et al., 2022). These findings indicate that green consumption may generate perceived social value by allowing individuals to view themselves as valued and meaningful members of society, thereby supporting emotional recovery under loneliness. Based on this reasoning, we hypothesize the following:

**H3.**  *Among individuals exposed to loneliness, engagement in green consumption increases perceived social value, which in turn enhances emotional recovery.*

## 3. Methodology

### 3.1. Preliminary Study

#### 3.1.1. Participants

In this study, loneliness was experimentally manipulated to investigate its impact on emotional recovery. Two participants who did not follow the instructions were excluded from the analysis, resulting in a final sample of one hundred and twenty-eight participants (93 females, M_age_ = 40.40, SD = 14.33) from around the world, recruited through the online survey platform Prolific. Sixty-four participants (45 females, M_age_ = 39.61, SD = 14.37) were randomly assigned to the loneliness priming condition, and sixty-four participants (48 females, M_age_ = 41.24, SD = 14.30) were randomly assigned to the control condition. We used G*Power 3.1 (power = 0.80, α = 0.05) to calculate that a minimum of one hundred and twenty-six participants was required, and the sample size we recruited satisfied this requirement.

#### 3.1.2. Method

The study was conducted in accordance with ethical guidelines, and all participants provided informed consent prior to participation. Before the task, participants rated their current emotional state on a 7-point scale (T1) (Cronbach’s alpha = 0.88) using items ranging from “not at all happy” to “very happy” and “not at all satisfied” to “very satisfied”, following the procedure adopted from previous studies (Gulledge et al., 2003; J. C. Lee et al., 2018; Tezer & Bodur, 2020). Participants were randomly assigned to one of two loneliness priming conditions. In the control condition, participants read the following message: “Think about a time when you felt that others valued you and appreciated your company or your contributions. You are rich in interpersonal relationships and have many friends who can support you when needed.” They were then asked to write about a specific instance in which they felt socially connected. In the loneliness priming condition, participants read, “Think about a time when you felt that others did not value you and did not appreciate your company or your contributions. No one understands you. You have no one to talk to.” They were then asked to write about a time when they felt socially disconnected. This manipulation closely follows the established loneliness priming paradigm employed in prior studies (Loughran Dommer et al., 2013; Tezer & Bodur, 2020; Yin & Lee, 2023).

After writing the essays, participants reported their emotional state again (T2) (Cronbach’s alpha = 0.94) to assess emotional recovery from loneliness. This procedure allowed us to examine within-subject changes in emotional states and test whether loneliness priming decreased participants’ current emotional state. At the end of the study, participants completed a loneliness manipulation check adopted from Jiao and Wang (2018), rating the extent to which they felt lonely and disconnected (Cronbach’s alpha = 0.96). All items were measured on a 7-point scale (1 = *not at all*, 7 = *extremely*), and participants also provided demographic information.

#### 3.1.3. Results

A manipulation check confirmed that perceived loneliness was higher in the loneliness priming condition (M = 5.47, SD = 1.43) compared to the control condition (M = 3.21, SD = 1.77; *F* (1, 127) = 62.79, *p* < 0.001, $\eta_{p}^{2}$ = 0.333). The emotional states across the two conditions are presented as follows. Prior to the experiment, there were no differences in T1 between the conditions (M_control_ = 3.84 [SD = 1.23], M_loneliness_ = 3.94 [SD = 1.20]; *F* (1, 127) = 0.19, *p* > 0.60, $\eta_{p}^{2}$ = 0.002). However, after the experimental manipulation, there was a difference in T2 (M_control_ = 5.19 [SD = 1.48], M_loneliness_ = 2.38 [SD = 0.97]; *F* (1, 127) = 162.10, *p* < 0.001, $\eta_{p}^{2}$ = 0.542).

We also conducted an ANCOVA with T2 as the dependent variable, loneliness as the independent variable, and T1 as a covariate to investigate recovery from loneliness. Results demonstrated that the main effects of loneliness type (*F* (1, 127) = 196.74, *p* < 0.001, $\eta_{p}^{2}$ = 0.611) on T2 were significant. Participants in the loneliness priming condition exhibited lower emotional recovery than those in the control condition, providing support for H1.

#### 3.1.4. Discussion

The findings indicate that experimentally induced loneliness significantly impairs emotional recovery, even after accounting for individuals’ baseline emotional states. Participants exposed to loneliness priming reported markedly lower post-task emotional well-being than those in the control condition, supporting prior research showing that loneliness depletes emotional resources and hinders affective restoration (Tezer & Bodur, 2020; Yin & Lee, 2023). These results establish loneliness as a critical emotional vulnerability, providing a foundation for examining conditions under which subsequent behaviors, such as green consumption, may facilitate emotional recovery.

### 3.2. Study 1

#### 3.2.1. Participants

This study employed a product experience task to delineate the boundary conditions of the effect of loneliness on emotional recovery from loneliness. The experiment used a 2 (product type: traditional vs. green) × 2 (loneliness: high vs. control) between-participants design and consisted of two parts. Participants were recruited from U.S. adults via the survey platform Prolific. Six participants were excluded for failing to follow instructions and not completing sufficient written vignettes, resulting in a final sample of one hundred seventy-nine participants (94 females, M_age_ = 42.87, SD = 13.48) included in the analyses. Ninety-two participants (52 females, M_age_ = 43.80, SD = 13.33) were randomly assigned to the loneliness priming condition, and eighty-seven participants (42 females, M_age_ = 41.89, SD = 13.65) were randomly assigned to the control condition. We used G*Power (power = 0.80, f = 0.25, α = 0.05) and calculated that a minimum of one hundred seventy-nine participants was required, confirming that the recruited sample size was sufficient to detect the expected effects.

#### 3.2.2. Method

The first part of the experiment involved loneliness manipulation, following the same procedure as in the preliminary study, in which participants wrote about a time they felt either socially connected or disconnected. In the second part, participants were asked to imagine a situation in which they would purchase a pen. In the green product condition, participants were informed that the pen was environmentally friendly (better for the environment), whereas in the traditional product condition, no such information was provided, consistent with the research design of Tezer and Bodur (2020). Participants then wrote an essay for one minute describing how and when they would use the item. Participants’ overall emotional state was assessed both before (T1) (Cronbach’s alpha = 0.94) and after (T2) (Cronbach’s alpha = 0.96) the essay task, as in the previous study. At the end of the experiment, participants completed a loneliness manipulation check (Cronbach’s alpha = 0.95) and provided demographic information.

#### 3.2.3. Results

A manipulation check revealed that perceived loneliness was higher in the loneliness condition (M = 5.64, SD = 1.65) compared to the control condition (M = 2.29, SD = 1.58; *F* (1, 178) = 190.67, *p* < 0.001, $\eta_{p}^{2}$ = 0.519). The participants’ emotional states in the two conditions were analyzed. There was no difference in their emotional state between the two conditions: before (T1) (M_control_ = 4.92 [SD = 1.43], M_loneliness_ = 4.69 [SD = 1.58]; *F* (1, 178) = 1.04, *p* > 0.30, $\eta_{p}^{2}$ = 0.006) and after (T2) the experiment (M_control_ = 4.91 [SD = 1.42], M_loneliness_ = 4.76 [SD = 1.62]; *F* (1, 178) = 0.45, *p* > 0.50, $\eta_{p}^{2}$ = 0.003).

**Figure 1:**
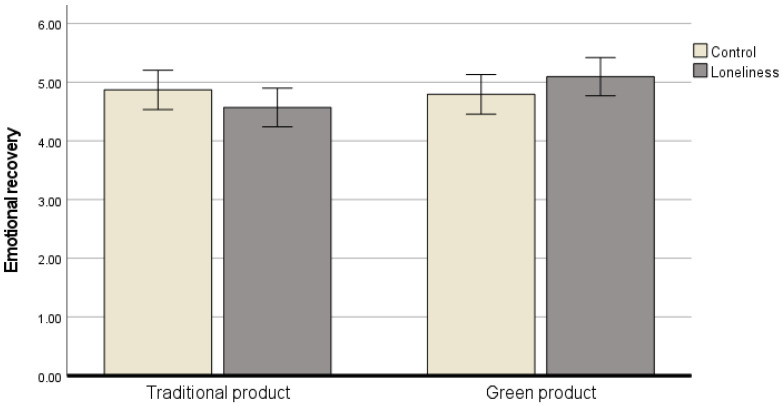
Interaction effects of loneliness and green consumption on emotional recovery.

We performed an ANCOVA with T2 as the dependent variable, loneliness and product type as the independent variables, and T1 as the covariate to examine the emotional recovery from loneliness. Results demonstrated the main effects of loneliness (*F* (1, 178) = 0.01, *p* > 0.90, $\eta_{p}^{2}$ = 0.000) and product type (*F* (1, 178) = 1.77, *p* > 0.10, $\eta_{p}^{2}$ = 0.010) were insignificant, but the interaction effect of loneliness and product type (*F* (1, 178) = 3.21, *p* < 0.10, $\eta_{p}^{2}$ = 0.018) on T2 was marginally significant. In the loneliness priming condition, participants who purchased green products reported significantly higher emotional recovery than those who did not (M_green_ = 4.89, SD = 1.53 vs. M_non-green_ = 4.62, SD = 1.70; *F* (1, 91) = 4.17, *p* < 0.05, $\eta_{p}^{2}$ = 0.045); however, considering participants in the control condition, there was no difference (M_green_ = 4.80, SD = 1.26 vs. M_non-green_ = 5.02, SD = 1.57; *F* (1, 86) = 0.12, *p* > 0.70, $\eta_{p}^{2}$ = 0.001) (Figure 1). These results indicate that purchasing green products facilitated emotional recovery among individuals in the loneliness priming condition.

#### 3.2.4. Discussion

The results further support the boundary condition of green consumption in alleviating the emotional consequences of loneliness. Specifically, lonely participants reported greater emotional recovery after engaging with a green product compared to a traditional product, whereas no such effect emerged in the control condition. This pattern provides preliminary support that green consumption functions as a situational coping mechanism that becomes salient under conditions of emotional vulnerability. Consistent with prior research emphasizing the symbolic and psychological well-being of sustainable consumption (Tezer & Bodur, 2020; Nguyen & Johnson, 2020), these findings indicate that engagement with green products can help restore emotional well-being among lonely individuals. Although the interaction effect between loneliness and product type was marginally significant in this study, participants in the loneliness priming condition still showed a significant restoration of emotional recovery through green consumption. These findings show that green consumption can mitigate the negative impact of loneliness on emotional recovery. Given that the attributes of eco-friendly products may have unintended consequences on environmental pollution intensity, the next study tested our hypothesis using dinnerware sanitizer, a product category in which consumers tend to perceive eco-friendly options more strongly. Therefore, the next study aims to investigate not only the effect of loneliness and green consumption on emotional recovery but also the mechanisms driving this effect.

### 3.3. Study 2

#### 3.3.1. Participants

We aimed to determine whether individuals exposed to the loneliness priming condition (vs. the control condition) would exhibit a higher emotional recovery when engaging in green consumption through social value. This study employed a 2 (product type: traditional vs. green) × 2 (loneliness: high vs. control) between-participants design and was conducted in two phases. Participants were U.S. adults recruited through the Prolific survey platform. Eight participants were excluded for failing to follow instructions or not completing sufficient written vignettes, resulting in a final sample of two hundred two participants (103 females, M_age_ = 44.81, SD = 13.70) included in the analyses. One hundred participants (52 females, M_age_ = 45.81, SD = 14.40) were randomly assigned to the loneliness condition, and one hundred two participants (51 females, M_age_ = 43.83, SD = 12.97) were randomly assigned to the control condition. We used G*Power 3.1 (power = 0.80, f = 0.25, α = 0.05) and calculated that a minimum of one hundred ninety-six participants was required, and the sample size we recruited satisfied this requirement.

#### 3.3.2. Method

In the first phase of the experiment, participants were asked about their current emotional state. Participants were then randomly assigned to either the loneliness or the control group. Using only one type of loneliness priming could raise concerns about the generalizability of our findings. To address this issue, in this study, we deliberately modified and extended the research design of Twenge et al. (2007) to test whether the proposed effects would replicate under a different operationalization of loneliness. Specifically, we adapted their paradigm to capture a broader experience of social exclusion as a form of loneliness, which is theoretically closely related to loneliness but instantiated in a different situational context.

Participants in the control condition were told, “Based on your responses above, I have good news for you—everyone thinks of you as someone they’d like to work with.” Participants in the loneliness priming condition, on the other hand, were told, “Based on your responses above, I hate to tell you this, but no one thinks of you as someone they want to work with.” They were then asked to write about a time when they felt socially accepted or rejected. The experimental design followed and modified the previous study (Twenge et al., 2007).

In the second phase of the experiment, participants were asked to imagine purchasing a dinnerware sanitizer. In the green product condition, participants were informed that the item was environmentally friendly, whereas in the traditional product condition, no such information was provided. Participants were then asked to write a brief essay explaining why they would choose either green or traditional products. This manipulation was adopted from the research of Tezer and Bodur (2020). Participants’ overall emotional state was measured both before (T1) (Cronbach’s alpha = 0.93) and after (T2) (Cronbach’s alpha = 0.95) the essay task, following the procedure used in Study 1. At the end of the experiment, participants completed the assessment of their social value with four items (e.g., “The product would help me to feel acceptable”, “The product would improve the way I am perceived”, “The product would make a good impression on other people”, and “The product would bring social approval from others”) (Cronbach’s alpha = 0.97), adopted from Sweeney and Soutar (2001); warm glow with bipolar pairs of semantic items (e.g., ashamed/proud, in the wrong/in the right, wicked/virtuous, and unethical/ethical) (Cronbach’s alpha = 0.95), adopted from Giebelhausen et al. (2016); green consumption manipulation check with two items (e.g., “I identified that the product I purchased is green.” and “I distinguished the product I purchased as green rather than traditional”) (Cronbach’s alpha = 0.94), adopted from Aitken et al. (2020) and Dhir et al. (2021); and a loneliness manipulation check with four items (Cronbach’s alpha = 0.99), providing demographic information consistent with the previous studies.

#### 3.3.3. Results

A manipulation check revealed that perceived green product was higher in the green product condition (M = 6.08, SD = 0.92) compared to the traditional product condition (M = 2.45, SD = 1.54; *F* (1, 201) = 416.23, *p* < 0.001, $\eta_{p}^{2}$ = 0.675). It also revealed that perceived loneliness was higher in the loneliness priming condition (M = 6.06, SD = 1.16) compared to the control condition (M = 1.95, SD = 1.11; *F* (1, 201) = 665.46, *p* < 0.001, $\eta_{p}^{2}$ = 0.769). The emotional states under the two conditions were assessed, and there was no difference in the emotional state between the two conditions, i.e., before (M_control_ = 4.71 [SD = 1.50], M_loneliness_ = 4.91 [SD = 1.67]; *F* (1, 201) = 0.76, *p* > 0.30, $\eta_{p}^{2}$ = 0.004) and after the experiment (M_control_ = 4.80 [SD = 1.64], M_loneliness_ = 4.91 [SD = 1.64]; *F* (1, 201) = 0.21, *p* > 0.60, $\eta_{p}^{2}$ = 0.001).

**Figure 2:**
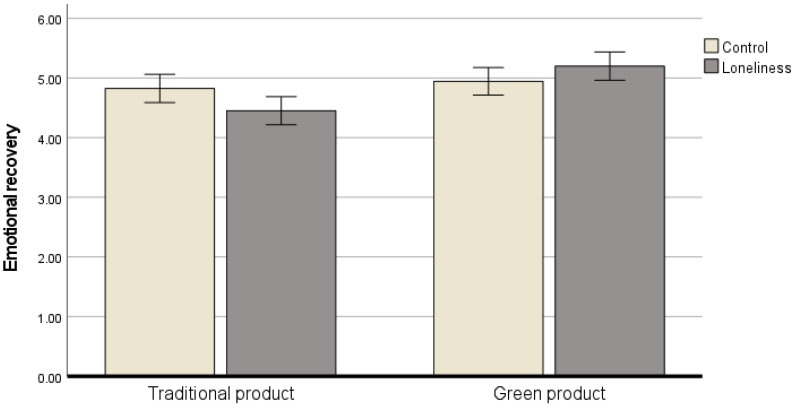
Interaction effects of loneliness and green consumption on emotional recovery.

ANCOVA with loneliness and product type as independent variables, the emotional recovery from loneliness (T2) as the dependent variable, and T1 as the control variable showed that the main effect of loneliness (*F* (1, 201) = 0.25, *p* > 0.60, $\eta_{p}^{2}$ = 0.001) was insignificant, but the main effect of product type (*F* (1, 201) = 13.12, *p* < 0.001, $\eta_{p}^{2}$ = 0.062) and interaction effect (*F* (1, 201) = 6.94, *p* < 0.01, $\eta_{p}^{2}$ = 0.034) were significant. In the loneliness priming condition, the emotional recovery was significant between participants who purchased the green product (M = 5.34, SD = 1.54) and those who purchased the traditional product (M = 4.48, SD = 1.63; *F* (1, 99) = 16.97, *p* < 0.001, $\eta_{p}^{2}$ = 0.149). However, in the control condition, the emotional recovery was insignificant between participants who purchased the green product (M = 5.09, SD = 1.60) and those who purchased the traditional product (M = 4.51, SD = 1.63; *F* (1, 101) = 0.16, *p* > 0.60, $\eta_{p}^{2}$ = 0.002) (Figure 2). That is, the decreased emotional state of individuals in the loneliness priming condition was restored when they engaged in green products.

Finally, to test whether social value mediated the effect of loneliness and product type on emotional recovery, after controlling for T1, we conducted a moderated mediation analysis using model 8 in PROCESS MACRO with 5000 bootstrapped samples (Hayes, 2017). The results revealed a significant index of moderated mediation (β = 0.26, SE = 0.10, 95% CI = [0.10, 0.47]). Specifically, the indirect effect through social value was significant in the loneliness priming condition (β = 0.33, SE = 0.10, 95% CI = [0.16, 0.55]) but not in the control condition (β = 0.08, SE = 0.05, 95% CI = [−0.01, 0.17]).

We reconducted the moderated mediation analysis with warm glow as a mediator. The mediation effect of warm glow (β = 0.08, SE = 0.08, 95% CI = [−0.07, 0.26]) was insignificant; therefore, the alternative mediation of warm glow can be excluded.

#### 3.3.4. Discussion

These findings align with prior research highlighting the psychological and social significance of consumption. Consistent with previous studies, green consumption can affirm one’s social value (Koller et al., 2011; Shrum et al., 2023), and we further found that sustainable consumption can facilitate emotional restoration under conditions of loneliness. Extending this perspective, our study responds to calls to examine the psychological foundations of environmentally responsible consumption (Nguyen & Johnson, 2020) by demonstrating that green consumption enhances emotional recovery, specifically through perceived social value. Together, these findings suggest that green consumption is not only environmentally meaningful but also socially and emotionally consequential, particularly for individuals experiencing social vulnerability.

## 4. Conclusions

The main aim of this study was to examine whether green consumption can serve as a coping strategy that alleviates the adverse impact of loneliness on emotional recovery. Across two studies, we first demonstrated that loneliness significantly undermines individuals’ capacity for emotional recovery. This finding is consistent with Caple et al. (2023), who highlight that individuals experiencing loneliness tend to show reduced recovery outcomes and diminished emotional well-being. One possible explanation is that loneliness constitutes a form of psychological distress that is closely associated with maladaptive cognitions and risky behavioral tendencies (Moore & Schultz, 1983; Shrum et al., 2023). Such distress may exhaust individuals’ emotional resources, making it more difficult for them to rebound from negative affective experiences and regain emotional stability.

Although prior research suggests that prosocial acts may reduce loneliness by fostering connection and belonging (Snyder & Newman, 2019; Fan et al., 2024; Y. Lee et al., 2024), green consumption has rarely been treated as a distinct coping strategy through which lonely individuals regulate negative emotions. Importantly, loneliness represents a special case in which individuals may be reluctant to engage in socially demanding prosocial behavior, as such interactions can heighten perceived social threat and discomfort (Yin & Lee, 2023). Extending this perspective, our study identifies green consumption as a unique form of prosocial engagement that does not require direct interpersonal involvement, yet it remains symbolically associated with social belonging (Malon et al., 2024). Our findings show that lonely individuals exhibit higher emotional recovery when engaging in green consumption, indicating that such everyday marketplace behavior may provide an accessible and low-risk means of alleviating loneliness-related distress. In this way, our results underscore that emotional recovery does not always depend on direct social engagement, which may sometimes increase social burden and stress for lonely individuals, but can also be supported through actions embedded in daily life.

Moreover, the present research clarifies the psychological mechanism underlying this effect by revealing perceived social value as a key mechanism through which green consumption facilitates emotional recovery among lonely individuals. Lonely individuals experienced greater emotional recovery when engaging in green consumption, not because such behavior involved direct interpersonal contact, but because it provided a sense of social acceptance, recognition, and approval. In other words, green consumption allowed individuals to feel socially valued even in the absence of immediate interaction. However, this mechanism also highlights an important boundary condition. While socially valued consumption may facilitate emotional recovery, simply relying on consumption as a mood-regulation strategy may become maladaptive when driven primarily by extrinsic motives such as materialistic value or status signaling. In contrast, intrinsically motivated green consumption grounded in genuine environmental concern may yield more enduring psychological benefits (Dittmar, 2005; Ridgway et al., 2008; Ryan & Deci, 2000). Nevertheless, our findings align with prior work suggesting that sustainable consumption carries a social meaning that enhances perceptions of respect and social recognition (Guo et al., 2020; Nguyen & Johnson, 2020; Tezer & Bodur, 2020).

Importantly, our results extend this literature by demonstrating that such socially valued meaning is particularly relevant in the context of loneliness-related recovery. When belongingness needs are threatened, individuals may rely on social value to restore a sense of connection. Supporting this interpretation, J. Li et al. (2022) show that belongingness needs a strengthening social identity and functioning, underscoring the psychological importance of feeling valued. Similarly, Hackel et al. (2017) suggest that prosocial behaviors generate emotional rewards partly because they affirm one’s social standing and identity, which aligns with our finding that eco-friendly purchasing can serve as a socially affirming act. Our findings suggest that this mechanism may be especially beneficial under conditions of psychological distress. Ying et al. (2022) demonstrate that social value orientation shapes how stress influences prosocial engagement, indicating that socially meaningful action can function as a coping resource. In line with this perspective, our study shows that green consumption offers lonely individuals an accessible way to regain perceived social value and emotional reassurance.

Unlike previous studies that highlight social integration as the primary route through which prosocial behavior alleviates loneliness (Fan et al., 2024; Y. Lee et al., 2024), our findings demonstrate that green consumption can promote emotional recovery through a more symbolic method by strengthening perceived social value. This enhanced sense of social value functions as a psychological resource that enables lonely individuals to feel emotionally reassured even without face-to-face interaction. Consequently, our study highlights that everyday sustainable consumption may serve not only environmental goals but also as a coping mechanism that supports resilience and emotional recovery under loneliness.

### 4.1. Theoretical Implications

The present findings offer several theoretical implications for research on loneliness recovery, prosocial behavior, and sustainable consumption. Although loneliness is widely understood as a distressing psychological state rooted in unmet belongingness needs (Baumeister & Leary, 1995; Hawkley & Cacioppo, 2010), prior work has primarily emphasized interpersonal reconnection and direct social engagement as the dominant strategy through which emotional relief is achieved. Our results challenge this interpersonal assumption by demonstrating that loneliness recovery may also unfold through indirect routes embedded in everyday consumer behavior.

In particular, eco-friendly purchasing may represent a low-risk and autonomous form of prosocial engagement through which individuals can regain a sense of social belonging, even when socially demanding behavior is less accessible (Lanser & Eisenberger, 2023; Fan et al., 2024). This insight extends models of loneliness regulation by highlighting that the coping process need not rely exclusively on face-to-face interaction but may also operate through marketplace behaviors that allow individuals to express contribution and affiliation in socially acceptable ways.

Moreover, our findings underscore that green consumption can generate psychological benefits that extend beyond its environmental outcomes (Gonçalves et al., 2016). Rather than functioning solely as an ethical marketplace choice, sustainable purchasing may serve as a meaningful coping strategy that reinforces purpose and self-perception. This interpretation aligns with prior work showing that meaningful consumption enhances social approval and positive self-regard (Mazar & Zhong, 2010; Gao & Mattila, 2016; White et al., 2019), strengthens perceived meaning in life (Tam & Chan, 2019), and alleviates feelings of loneliness (Wang et al., 2021). Hence, sustainable consumption should be more centrally integrated into broader theoretical frameworks of emotional coping and well-being.

Finally, the mediating role of perceived social value highlights social approval and recognition as a critical psychological mechanism linking green consumption to emotional recovery. Green consumption appears to operate not only through moral or instrumental benefits but also through symbolic affirmation, enabling individuals to feel recognized and socially valued despite relational disconnection. In doing so, our findings extend consumer psychology into wider theoretical discussions of belongingness and resilience, suggesting that emotional recovery from loneliness may be supported through socially meaningful actions embedded in daily life.

### 4.2. Practical Implications

Managers should recognize that consumers differ in their levels of social connectedness and that individuals experiencing loneliness often face distinct psychological barriers, including heightened sensitivity to rejection and discomfort with socially demanding interactions. Accordingly, interventions aimed at supporting such consumers should not rely exclusively on promoting direct social contact but should also provide emotionally safe, low-pressure avenues through which individuals can regain a sense of connection.

Our findings suggest that green consumption can serve as one such avenue. Because eco-friendly purchasing allows consumers to perceive themselves as socially valued and meaningful existences without requiring immediate interpersonal engagement, firms can position green products not only as environmentally responsible choices but also as psychologically supportive ones. Marketing communications may therefore emphasize how sustainable actions allow consumers to feel aligned with shared values and meaningful purpose, even in moments of social disconnection.

From a behavioral science perspective, green purchasing may operate as a form of symbolic prosocial participation, offering consumers an indirect way to express care and social contribution. Firms can strengthen this restorative potential by embedding clearer social value cues into sustainable branding, such as highlighting meaningful contributions, shared responsibility, or quiet forms of participation. For example, advertisements may frame eco-friendly choices as small but valuable acts that connect individuals to something larger than themselves, rather than relying solely on moral appeals. Examples include phrases such as “Even small green choices connect us to something bigger” or “Your care for the environment reflects the kind of person society values.” Such approaches highlight connection and contribution; such approaches may resonate particularly with consumers experiencing perceived disconnection, thereby positioning sustainable products as supportive choices that promote emotional reassurance. Ultimately, these strategies suggest that sustainability marketing can be aligned not only with environmental objectives but also with consumer well-being.

### 4.3. Limitations

While the present study identifies perceived social value as a key mechanism through which green consumption promotes emotional recovery among lonely individuals, other potential mechanisms may also contribute to this process. Future research could explore additional psychological or motivational factors, such as prosocial motivation or self-efficacy, that may further explain how environmentally responsible behaviors facilitate emotional recovery from loneliness. Moreover, testing these mechanisms across broader and more diverse populations would help to enhance the generalizability of the findings and provide a more comprehensive understanding of how green consumption contributes to emotional recovery.

Although prior research has employed social exclusion scenarios, such as the Cyberball game, to examine the effects of social disconnection or loneliness, we did not include this type of experiment in the present study. In future research, such scenarios could be incorporated to further investigate how green consumption may buffer the emotional consequences of loneliness, providing additional insights into the generalizability and robustness of our findings. Furthermore, larger samples are needed to boost power by increasing the observed effect size.

The final limitation of this research is that we did not include explicit suspicion checks regarding the feedback in Study 2. As a result, it is possible that some participants questioned the authenticity of the feedback, which may have implications for the study’s internal validity. Future studies could include suspicion-check questions or conduct a real-time feedback study to strengthen the validity of the findings.

## Data Availability

The data presented in this study are available upon request from the authors.
